# Chronotherapy as a novel strategy to limit anthracycline-induced cardiotoxicity

**DOI:** 10.1093/cvr/cvaf179

**Published:** 2025-10-06

**Authors:** Ilse R Kelters, Markella I Printezi, Annabelle Ballesta, Pieterjan Dierickx, Yvonne Koop, Pasquale F Innominato, Francis A Lévi, J Corné van Dam, Pieter A Doevendans, Alwin D R Huitema, Arco J Teske, Anne M May, Joost P G Sluijter, Linda W van Laake

**Affiliations:** Department of Cardiology and Experimental Cardiology Laboratory, Division of Heart & Lungs, University Medical Center Utrecht, Heidelberglaan 100, 3584 CX Utrecht, The Netherlands; Department of Cardiology and Experimental Cardiology Laboratory, Division of Heart & Lungs, University Medical Center Utrecht, Heidelberglaan 100, 3584 CX Utrecht, The Netherlands; Inserm Unit 900, Cancer Systems Pharmacology Team, Institut Curie, Saint-Cloud, France; Max Planck Institute for Heart and Lung Research, Bad Nauheim, Germany; Cardiopulmonary Institute (CPI), Bad Nauheim, Germany; German Center for Cardiovascular Research (DZHK), Partner Site Rhine-Main, Bad Nauheim, Germany; Julius Center for Health Sciences and Primary Care, Utrecht University Medical Center, Utrecht University, Utrecht, The Netherlands; Department of Oncology-Pathology, Karolinska Institutet, Stockholm, Sweden; Oncology Department, Ysbyty Gwynedd Hospital, Betsi Cadwaladr University Health Board, Bangor, UK; Cancer Research Center, Warwick Medical School, University of Warwick, Coventry, UK; Chronotherapy, Cancers and Transplantation Research Unit, Faculty of Medicine, Paris-Saclay University, Villejuif, France; Regenerative Medicine Center Utrecht, Circulatory Health Research Center, University Utrecht, Utrecht, The Netherlands; Department of Cardiology and Experimental Cardiology Laboratory, Division of Heart & Lungs, University Medical Center Utrecht, Heidelberglaan 100, 3584 CX Utrecht, The Netherlands; Department of Clinical Pharmacy, University Medical Center Utrecht, Utrecht University, Utrecht, The Netherlands; Department of Cardiology and Experimental Cardiology Laboratory, Division of Heart & Lungs, University Medical Center Utrecht, Heidelberglaan 100, 3584 CX Utrecht, The Netherlands; Department of Epidemiology, Julius Center for Health Sciences and Primary Care, Utrecht University, Utrecht, The Netherlands; Department of Cardiology and Experimental Cardiology Laboratory, Division of Heart & Lungs, University Medical Center Utrecht, Heidelberglaan 100, 3584 CX Utrecht, The Netherlands; Regenerative Medicine Center Utrecht, Circulatory Health Research Center, University Utrecht, Utrecht, The Netherlands; Department of Cardiology and Experimental Cardiology Laboratory, Division of Heart & Lungs, University Medical Center Utrecht, Heidelberglaan 100, 3584 CX Utrecht, The Netherlands; Regenerative Medicine Center Utrecht, Circulatory Health Research Center, University Utrecht, Utrecht, The Netherlands

**Keywords:** Circadian Rhythms, Chronotherapy, Chronomodulation, Anthracycline, Cardiotoxicity, Cardio-oncology

## Abstract

Anthracycline cardiotoxicity is a severe chemotherapeutic side effect that can lead to heart failure in cancer patients and survivors. Chronomodulated chemotherapy is a promising preventive strategy that encompasses the adjustment of anthracycline administration time to the circadian rhythms (24-hour rhythms) of the body. Circadian rhythms play a major role in cardiovascular physiology and disease and may lead to a time-dependent variation in cardiac sensitivity to anthracyclines. In this review, all available evidence on the topic of chronomodulated anthracyclines for cardiotoxicity reduction and/or oncological efficacy enhancement is summarized. In total, 3 *in vitro* studies, 12 animal studies, and 9 clinical studies were included. Potential mechanistic explanations involved 24-hour variation in oxidative stress regulation, DNA damage repair, and systemic or intracellular pharmacokinetics. We identified a hypothesized optimal time frame from 3 to 11 AM for anthracycline administration in humans, based on extrapolation of findings in animal studies.

## Graphical Abstract

**Graphical Abstract:**
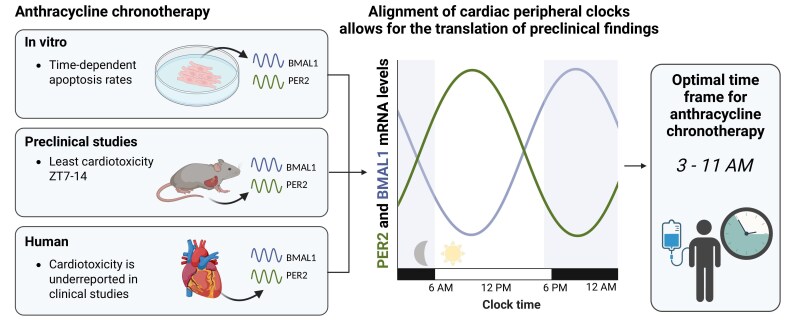

## 1. Introduction

### 1.1 Anthracyclines and cardiotoxicity

Anthracyclines are a group of chemotherapeutics that are commonly used against various cancers, such as breast cancer and haematological malignancies. Currently used agents include doxorubicin, daunorubicin, epirubicin, idarubicin, and the synthetically derived anthracenedione mitoxantrone. Unfortunately, their application is limited by their dose-dependent cardiotoxic effects, which lead to cardiac dysfunction ranging from an (sub)acute rise in serum levels of cardiac troponin (in almost 50% of patients,^1^ to clinically overt systolic dysfunction (at echocardiogram or nuclear scan) in approximately 6% of patients.^2^ Since anthracycline-induced cardiac damage is largely irreversible, the only therapeutic option currently consists of early detection of subclinical cardiotoxicity and attempting to delay deterioration into heart failure.^3^ When cardiac dysfunction deteriorates into heart failure, it has a significant impact on quality of life and severely limits life expectancy.^4^ Therefore, prevention of anthracycline-induced cardiac damage should be prioritized. Previous research has aimed to determine a heart-safe cumulative dose and has explored pharmacological strategies, such as liposomal anthracycline formulations, the concomitant use of dexrazoxane (an iron chelation agent), and heart failure medication. Although some of these strategies have shown promising effects in preliminary studies, their use remains limited to specific indications because of a lack of sufficient evidence.^5^

### 1.2 Chronomodulated chemotherapy

Chronomodulated chemotherapy, which involves adjusting drug administration time to align with the body’s 24-hour rhythms, is a promising approach for reducing cardiotoxicity. Following the awarding of the Nobel Prize in Physiology or Medicine in 2017 for groundbreaking discoveries in molecular chronobiology, interest in the applications of circadian rhythms has grown rapidly, especially in oncology.^6^ Circadian rhythms (*circa =* around, *dies* = day) encompass the intrinsic 24-hour oscillations in physiological functions and are generated by ubiquitous genetic clocks.

**Figure 1:**
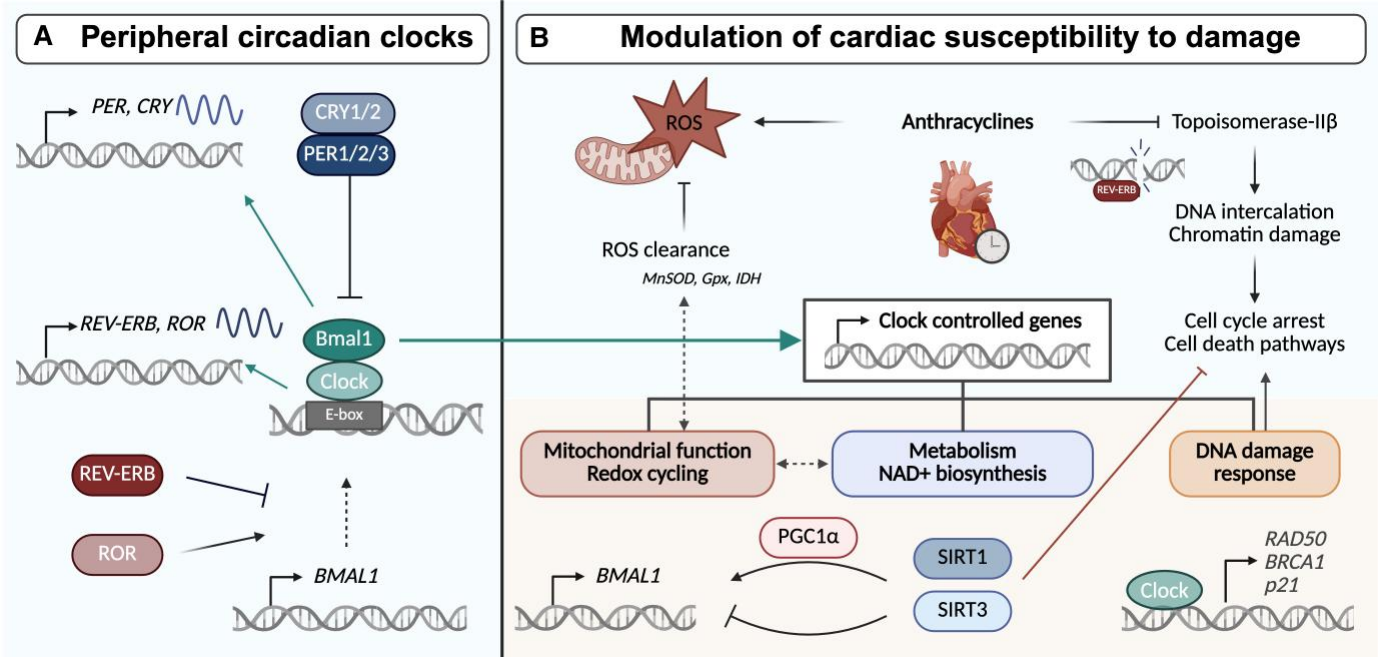
Molecular interaction between the circadian core clock and key cellular processes affecting cardiac susceptibility to damage. (*A*) The key components of these feedback loops are BMAL1 and CLOCK, two proteins that form a heterodimeric complex that activates the transcription of the cryptochrome (*CRY*) and period (*PER*) genes. The resulting CRY and PER proteins dimerize and inhibit the activating function of CLOCK-BMAL1, creating a feedback loop.^10–12^ Another loop involves the circadian nuclear receptors REV-ERBα and REV-ERBβ, and retinoic acid receptor-related orphan receptors RORα, RORβ, and RORγ which suppress and activate *BMAL1* and *CLOCK* expression, respectively.^13–15^ (*B*) The cardiomyocyte circadian clock modulates this susceptibility by regulating metabolic and stress-response pathways. Created in Biorender.

In mammals, the suprachiasmatic nucleus in the hypothalamus, also referred to as the ‘central pacemaker’, generates 24-hour oscillations and is synchronized to the environment principally through light. This central pacemaker in turn is responsible for the coordination of peripheral clocks, which are present in most cells of the body. In addition, peripheral clocks are synchronized by specific external cues (‘*Zeitgebers*’), such as the alternation of eating and fasting, that of rest and activity, and temperature cycles.^7^ The peripheral cellular clocks are composed of self-sustaining intertwined transcriptional and (post-) translational feedback loops involving core clock genes (e.g. *CLOCK*, *BMAL1*, *PER1-3*, and *CRY1-2*) (*Figure 1A*).^8,9^ This hierarchical network system temporally coordinates physiological functions at cellular, tissue, and whole organism levels. Peripheral clock machinery has been found in every cardiovascular cell type, such as cardiomyocytes, cardiac fibroblasts, and endothelial cells.^16^ These peripheral clocks in the heart may give rise to a diurnal variation in cardiac sensitivity to anthracyclines.

### 1.3 Circadian modulation of cardiac susceptibility to damage

The cardiotoxic and anticancer properties of anthracyclines are mechanistically intertwined. These drugs exert their antitumour effect via the inhibition of topoisomerase-II, DNA intercalation, and chromatin damage, resulting in cell cycle arrest and induction of cell death pathways, which also contribute to off-target toxicity in cardiomyocytes (*Figure 1B*).^17,18^ Additionally, anthracyclines generate reactive oxygen species (ROS) through mitochondrial dysfunction and redox cycling, to which cardiomyocytes, due to the high mitochondrial content,are particularly susceptible.^19^

The cardiomyocyte circadian clock modulates this susceptibility by regulating metabolic and stress-response pathways.^20–22^ Central to this regulation are the NAD^+^-dependent deacetylases Sirtuin 1 (SIRT1) and Sirtuin 3 (SIRT3), which are subject to circadian control through the CLOCK-BMAL1 complex and its interaction with NAD^+^ biosynthesis.^23,24^ SIRT1 indirectly regulates *BMAL1* expression via coactivation of PGC1α, while SIRT3 deacetylates BMAL1, reducing CLOCK-BMAL1 activity and creating a negative feedback loop.^23,25,26^ Both SIRT1 and SIRT3 deacetylate CLOCK-BMAL1 target genes and influence diverse metabolic processes, including the enhancement of fatty acid oxidation and improvement of mitochondrial function.^27–29^ The presence of NAD^+^ during the catabolic phase leads to a peak in ROS clearance, driven by manganese superoxide dismutase (MnSOD), glutathione peroxidase (GPx), and isocitrate dehydrogenase (IDH).^27,30^ In addition, overexpression of SIRT3 mitigates doxorubicin-induced mitochondrial dysfunction and ROS production,^31,32^ while inhibition of SIRT3 increases the apoptosis rate in doxorubicin-treated human induced pluripotent stem cell-derived cardiomyocytes.^32^ Thus, the circadian regulation of myocardial oxidative stress levels through SIRT1 and SIRT3 may offer a partial mechanistic explanation for 24-hour variations in anthracycline cardiotoxicity.

In addition to modulating oxidative stress, circadian clock components also regulate DNA damage response. CLOCK transcriptionally activates genes involved in DNA double-strand break repair, including *RAD50*, *BRCA1*, and *p21*.^33^ PER2 affects the expression of *p53* target genes by modulating p53, while nuclear receptor REV-ERBα (also known as nuclear receptor subfamily 1, group D, member 1, NR1D1) directly regulates *p21* on a transcriptional level and binds to double-strand breaks, hindering the successful recruitment of the repair machinery.^34–36^ Although cardiac topoisomerase-IIβ does not exhibit transcriptional circadian oscillating patterns in human cardiac tissue, this does not rule out possible circadian protein activity due to post-transcriptional regulation.^37,38^ Together, these findings suggest that both mitochondrial redox balance and DNA repair capacity are subject to circadian modulation, providing a plausible mechanistic framework for time-of-day-dependent differences in anthracycline cardiotoxicity.

### 1.4 Rationale and objectives

While there is insufficient clinical data exploring chronomodulated chemotherapy for cardiotoxicity reduction, several trials have focused on other types of toxicities. A recent systematic review performed by our research group, including 18 randomized controlled trials (RCTs), revealed that chronomodulated chemotherapy led to a toxicity reduction in most trials, while efficacy was maintained in all studies, as compared to non-circadian-based protocols.^39^ To develop a better understanding of the potential of chronomodulated anthracyclines to reduce cardiotoxicity, a dedicated randomized controlled trial (RCT) investigating the effect of different administration timings would be essential. In designing such an RCT, the following potential influences on optimal chemotherapeutic timing should be considered: age, biological sex, chronotype, comorbidities, malignancy type and stage, and type of anthracyclines.^39–41^ Hence, to isolate the effect of anthracycline administration time, a homogenous study population would be needed, with an adequate sample size for detecting potential sex-related differences. Equally important, a solid theoretical framework is needed to substantiate the selected administration times in such a trial, ensuring that the hypothesized optimal time frame is included. Determining this time frame poses an intricate challenge since the current literature on this topic involves *in vitro* studies and data from nocturnal animal models, while human data is lacking. Therefore, in-depth consideration of the available literature is required to achieve adequate translation to the clinical setting. To this end, we conducted a comprehensive review, including preclinical and clinical studies that evaluated the toxicity and efficacy of chronomodulated anthracycline-based therapies. A systematic search of PubMed was performed using terms related to circadian rhythms, chronotherapy, and anthracyclines, without restriction on publication date (see Supplementary material online, *Table S1*). Studies were selected based on predefined eligibility criteria, and relevant data were extracted following a structured methodology (see Supplementary material online, *Figure S1*).

With this narrative review, we aim to provide a critical overview of the current literature on chronomodulated anthracycline administration as a strategy to reduce cardiotoxicity. Based on our findings, we will provide a hypothesized optimal anthracycline administration time frame, at which we expect cardiac sensitivity to anthracyclines to be lowest.

## 2. Overview of *in vitro* studies on chronomodulated anthracyclines

So far, three studies have investigated 24-hour variations in apoptotic response upon anthracycline exposure *in vitro,* using human embryonic stem cell-derived cardiomyocytes, neonatal rat cardiomyocytes, or human stem cell antigen 1-positive cells.^42–44^ Across all studies, cells revealed circadian stress-responsive behaviour when exposed to doxorubicin (10 μM for 6 h), with apoptosis rates being the highest between Circadian Time (CT) 18–27, and the lowest between CT36-45, with CT0 corresponding to synchronization onset (see Supplementary material online, *Table S2*). Interestingly, these findings correlated with several stress-response genes, such as *HSPH1*, *DNAJA1*, and *RRAGA*, that were identified as clock-controlled genes and displayed circadian oscillations in human embryonic stem cell-derived cardiomyocytes.^43^ This suggests that these stress-response genes may contribute to the time-dependent tolerance of cardiomyocytes to anthracyclines.

Previous research on the circadian clock in cancers, comparing cancerous vs. normal tissues, demonstrated that clock genes exhibit cancer-specific expression patterns across various cancer types.^45–47^ Some cancer cells possibly alter their intrinsic circadian clock by suppressing their clocks’ negative regulators (PER, CRY, and REV-ERB), while others demonstrate an increased dependence on the CLOCK-BMAL1 dimer.^46,48^ Additionally, the number of genes that correlate with the expression of clock genes is substantially lower in cancerous tissues than in normal tissues.^45^ Clock gene correlation analysis of paired cancerous and non-cancerous breast tissues from breast cancer patients, who are often treated with anthracyclines, revealed subtype-specific changes in rhythm magnitude.^49^ The impact of circadian clock alterations in cancers is specific to the tumour type and characteristics.^45^ Therefore, in some cancers, this may lead to a time-dependency in chemotherapeutic efficacy, while other cancers may be unaffected by administration time.

In conclusion, the abovementioned intricate molecular mechanisms highlight the dynamic interplay between circadian rhythms, metabolism, and DNA repair in cardiomyocytes. While *in vitro* studies have contributed valuable insights into the time-dependent vulnerability of cardiac cells to anthracyclines, it is crucial to recognize that these models exclusively investigate intrinsic cellular circadian regulation. They do not fully recapitulate the complexities of systemic oscillations mediated by both peripheral and central clocks that regulate organs and physiological systems over the 24-hour span. To expand our understanding, the next step involves exploring animal studies, which can provide a more holistic perspective on physiological responses to stressors according to administration timing.

## 3. Overview of animal studies on chronomodulated anthracyclines

### 3.1 Pharmacodynamic effects of anthracycline chronomodulation

**Figure 2:**
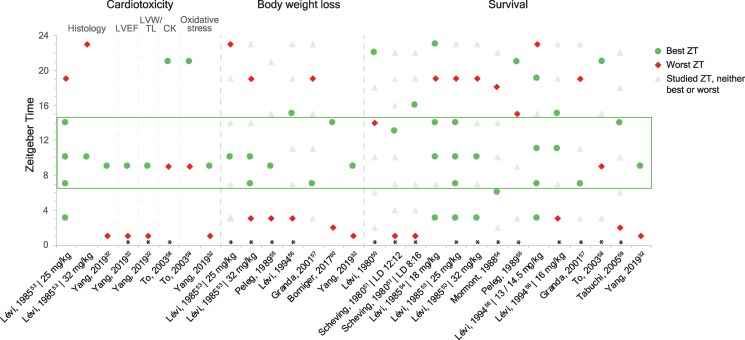
Overview of relevant findings on cardiotoxicity, body weight loss, and survival, from included animal studies in rodents. The rectangle indicates the time frame (between Zeitgeber Time (ZT) 7 and 14 in which anthracycline administration may be most favourable for reducing cardiotoxicity, minimizing body weight loss, and increasing survival. In case multiple follow-up periods were reported for survival in one study, the follow-up time closest to 60 days was plotted in this figure. LVEF, left ventricular ejection fraction; LVW/TL, left ventricular weight/tibial length. *Statistically significant difference (*P* < 0.05).

Research on the impact of circadian rhythms on alleviating chemotherapy-induced cardiotoxicity in animal studies is limited. Between 1979 and 2024, 12 studies were published on the circadian-based efficacy and toxicity profiles of anthracyclines, with three of them reporting cardiac outcomes (*Table 1*, *Figure 2*). First, Levi *et al.*^53^ studied cardiotoxicity-specific outcomes according to the time of pirarubicin administration over the 24-hour span in mice subjected to alternation of 12 h of light and 12 h of darkness (LD12:12), Zeitgeber Time (ZT) 0 being defined as light onset. Cardiac lesions in histopathological sections were only observed in the case of anthracycline administration during ZT19-23 (*Table 2*).^53^ Second, Yang *et al.*^32^ investigated the impact of the timing of doxorubicin administration on cardiotoxicity in rats, studying two administration times, ZT1 and ZT9, selected as the trough and peak of SIRT3 expression, respectively. Administering doxorubicin at ZT9 resulted in a better preservation of left ventricular ejection fraction (LVEF) and fractional shortening compared to ZT1.^32^ Congruently, administration at ZT9 reduced H_2_O_2_ levels and prevented inhibition of MnSOD activity in mitochondria.^32^ In contrast, To *et al.*^58^ reported no discernible impairment in the activity of the antioxidant defence system, as indicated by the GPx levels in myocardial tissue following chronomodulated administration of doxorubicin in rats. However, they observed significant elevations in myocardial lipid peroxide levels, along with increased serum creatine kinase levels, after doxorubicin administration at ZT9 in comparison to ZT21.^58^

**Table 1:** Baseline characteristics of included animal studies

First author, year	Species, sex, disease model	Age	Group (*n*=)	Total (*n*=)	ZT	Treatment regimen	Collected outcomes
Lévi *et al.*^50^	CD2F1 mice, F	5–7	24	144	2, 6, 10, 14, 18, and 22	18 mg/kg doxorubicin i.v. single shot	Survival rate
Scheving *et al.*^51^	C57BL/6J × DBA/2J F1 mice, M, LL	6	25	200	1, 4, 7, 10, 13, 16, 19, and 22	5 mg/kg doxorubicin^a^ + 100 mg/kg cyclophosphamide^a^ single shot	Survival rate
	CD2F1 mice, F, LL	6	18–21	120	1, 4, 7, 16, 19, and 22	5 mg/kg doxorubicin^a^ + 100 mg/kg cyclophosphamide^a^ single shot, 8:16 L:D	Survival rate
Halberg *et al.*^52^	Fisher rats, F, mammary adenocarcinoma	5	4–24	216	2, 6, 10, 14, 18, and 22	0.8 mg/kg doxorubicin i.p. daily for 7 days, followed by 1.6 mg/kg phenylalanine mustard p.o. 3 times per week, 8:16 L:D	Time to TR, TR duration, and total TR rate
Lévi *et al.*^53^	B6D2F1 mice, M	22	8–9	226	3, 7, 10, 14, 19, and 23	18, 25, and 32 mg/kg pirarubicin i.v. single shot	Cardiac histology, body weight, and survival rate
Mormont *et al.*^54^	CD2F1 mice, F	24	68–70	417	2, 6, 10, 14, 18, and 22	25 mg/kg epirubicin i.p. single shot	Survival rate
Peleg *et al.*^55^	BALB/c mice, M	26	30	120	3, 9, 15, and 21	1 mg/kg doxorubicin^a^ daily for 15 days, 14:10 L:D	Body weight and survival rate
Lévi *et al.*^56^	B6D2F1 mice, M	11	8	144	3, 7, 11, 15, 19, and 23	13, 14.5, or 16 mg/kg mitoxantrone i.v. single shot	Body weight and survival rate
Granda *et al.*^57^	C3H/HeN mice, M, mammary adenocarcinoma	5	8	333	3, 7, 11, 15, 19, and 23	3, 5, 8.3, or 13.8 mg/kg doxorubicin i.v. once a week for 3 weeks	Body weight, survival rate, complete TI, and duration of TI
To *et al.*^58^	Donryu rats, M	3	10	105	3, 9, 15, and 21	5 mg/kg doxorubicin i.p. once a week for 4 weeks	Serum CK, cardiac GPx, cardiac MDA, survival rate
Tabuchi *et al.*^59^	ICR mice, M	6	11	66	2, 6, 10, 14, 18, and 22	5 mg/kg doxorubicin i.p. once weekly for 4 weeks	Survival rate
Borniger *et al.*^60^	BALB/c mice, F	8	7 and 8	15	2 and 14	13.5 mg/kg doxorubicin i.v. +135 mg/kg cyclophosphamide i.v. single shot	Body weight
Yang *et al.*^32^	Sprague Dawley rats, M	NR	10–25	100	1 and 9	1.7 mg/kg doxorubicin i.v. once weekly for 6 weeks	LVEF, FS, H_2_O_2_, MnSOD, LVW/TL ratio, cardiac histology, body weight, survival rate

Age is shown in weeks. All studies used a light–dark schedule of 12:12, unless otherwise specified in the treatment regimen. The total animal count does not always equal the number of studied ZT multiplied by the number of animals per group, since some studies could not measure all outcomes in the same group of animals.

F, female; FS, fractional shortening; GPx, glutathione peroxidase; H_2_O_2_, hydrogen peroxide; i.p., intraperitoneal; i.v., intravenous; L:D, light–dark regimen; LL, lymphatic leukaemia; LVEF, left ventricular ejection fraction; LVW/TL, left ventricular weight/tibia length; M, male; MDA, malondialdehyde; MnSOD, manganese superoxide dismutase; NR, not reported; P.o., per os; TI, tumour growth inhibition; TR, tumour remission; ZT, zeitgeber time.

^a^Route of administration not reported.

**Table 2:** Summary of cardiotoxicity indicators at different ZTs of anthracycline administration

First author, year	Treatment	Studied ZTs	Cardiotoxicity indicator	Follow-up time	Best ZT	Worst ZT	Best vs. worst specification	Remarks
Levi *et al.*^52^	25 mg/kg pirarubicin i.v. single shot	3, 7, 10, 14, 19, and 23	Myocardial histology	20 days	(3, 7, 10, 14, 23)	19	Disarray in 0/12 vs. 2/2 mice	Significance level NR
	32 mg/kg pirarubicin i.v. single shot	10 and 23	Myocardial histology	20 days	10	23	Disarray in 0/4 vs. 1/4 mice	Significance level NR
To *et al.*^61^	20 mg/kg doxorubicin i.p. intermittent	9 and 21	Serum CK	14 days	21	9	+36.2% vs. +132.7%^a^	
				28 days	21	9	+32.4% vs. +134.2%^a^	
			Cardiac MDA	14 days	21	9	−8.3% vs. +29.3%^a^	
				28 days	9	21	−4.3% vs. +15.7%	
			Cardiac GPx	14 days	9	21	−12.9% vs. −6.9%	
				28 days	NA	NA	−22.4% vs. −22.1%	
Yang *et al.*^46^	10.2 mg/kg doxorubicin i.v. intermittent	1 and 9	LVEF	98 days	9	1	−9.6% vs. −31.1%^a^	
			FS	98 days	9	1	−16.7% vs.−43.5%^a^	
			LVW/TL	98 days	9	1	−16.7% vs. −22.2%^a^	
			Myocardial histology	98 days	9	1	Less disarray vs. more disarray^b^	Significance level NR
			H_2_O_2_	98 days	9	1	+17.5% vs. +49.6%	Significance level NR
			MnSOD	98 days	9	1	−4.7% vs. −41.3%	Significance level NR

All animals were exposed to a 12:12 light–dark regimen. Dose indicates cumulative dose. Specification should be interpreted as follows: percentual differences between the intervention group and the sham group at the same ZT. E.g. at ZT21, CK concentration was 36.2% higher in the doxorubicin-treated group compared to the sham group, and this was 132.2% for ZT9. An increase in the following parameters (hypothetically) corresponds with more cardiac damage: CK, MDA (increase in oxidative stress), and H2O2 (increase in oxidative stress). A decrease of the following parameters (hypothetically) corresponds with more cardiac damage: GPx (neutralizes oxidative stress), LVEF, FS, LVW/TL, and MnSOD (neutralizes oxidative stress).

CK, creatine kinase; FS, fractional shortening; GPx, glutathione peroxidase; H₂O₂, hydrogen peroxide; i.p., intraperitoneal; i.v., intravenous; LVEF, left ventricular ejection fraction; LVW/TL, left ventricular weight/tibia length; MDA, malondialdehyde; MnSOD, manganese superoxide dismutase; NR, not reported; ZT, zeitgeber time.

^a^Statistically significant difference (*P* < 0.05) represents the statistical comparison of values of the best vs. the worst timepoints.

^b^ZT1: Extremely disordered myocardial fibres, dissolved myofibrils, and swelling of myocardial cells. ZT9: limited cytoplasmic vacuolation, with or without myofibrillar loss. The number of evaluated samples was not reported.

In the abovementioned studies, the timing of least cardiotoxicity aligned with the time of best survival.^32,53,58^ Eight additional *in vivo* studies in rodents have primarily focused on the overall toxicity of anthracycline-based treatment as determined by body weight loss and/or survival rates (*Tables 3* and *4*). These organism-level endpoints are associated with overall drug side effects, including cardiotoxicity and bone marrow depression, while also reflecting treatment efficacy in tumour-bearing rodents. When directly comparing the results of these studies, the varying experimental conditions need to be considered.

**Table 3:** Summary of body weight results at different ZTs of anthracycline administration

First author, year	Treatment	Studied ZTs	Follow-up time	Best ZT	Worst ZT	Best vs. worst specification	Remarks
*Follow-up time < 1 week*							
Lévi *et al.*^53^	25 mg/kg pirarubicin i.v. single shot	3, 7, 10, 14, 19, and 23	5 days	10	23	−8.9% vs. −16.7%^a^	
	32 mg/kg pirarubicin i.v. single shot	3, 7, 10, 14, 19, and 23	5 days	7 + 10	3 + 19	−20.9% vs. −15.5%^a^	
Borniger *et al.*^60^	13.5 mg/kg doxorubicin + 135 mg/kg cyclophosphamide i.v. single shot	2 and 14	1 day	(14)	(2)	+1.68% vs. −1.76%	Significance level NR
*Follow-up time ≥ 1 week*							
Peleg *et al.*^55^	15 mg/kg doxorubicin intermittent. 14:10 LD	3, 9, 15, and 21	50 days	9	3	+2.4% vs. −14.9%^a^	Administration route NR
Lévi *et al.*^56^	13, 14.5 or 16 mg/kg mitoxantrone i.v. single shot	3, 7, 11, 15, 19, and 23	10 days	15	3	−32% vs. −41%^a^	
Granda *et al.*^57^	9–41.4 mg/kg doxorubicin i.v. intermittent	3, 7, 11, 15, 19, and 23	≥ 7 days	(7)	(19)	−1.7% vs. −8.5%	Nadir weight was reached at different follow-up times; Significance level NR; Cancer induced in animal model
Yang *et al.*^32^	10.2 mg/kg doxorubicin i.v. intermittent	1 and 9	50 days	9	1	+21.9% vs. +14.2%^b^	

Animals in all studies except one were exposed to a 12:12 light–dark regimen, only the study by Peleg et al. used a 14:10 light–dark regimen. Dose indicates cumulative dose. Specification should be interpreted as follows: percentages show the change compared to the pre-treatment weight.

I.v., intravenous; L:D, light–dark regimen; NR, not reported; ZT, zeitgeber time.

^a^Statistically significant difference (*P* < 0.05) represents the statistical comparison of values of best vs. worst timepoints.

^b^Difference in weight between days 49–84 was statistically significant, favouring ZT 9.

**Table 4:** Summary of survival at different ZTs of anthracycline administration

First author, year	Treatment	Studied ZTs	Follow-up time	Best ZT	Worst ZT	Best vs. worst specification	Remarks
Lévi *et al.*^50^	18 mg/kg doxorubicin i.v. single shot	2, 6, 10, 14, 18, and 22	64 days	22	14	63.6% vs. 19.0%^a^	
			84 days	(18)	(10)	27.3% vs. 4.8%	
Scheving *et al.*^51^	5 mg/kg doxorubicin + 100 mg/kg cyclophosphamide single shot. 12:12 L:D	1,4, 7, 10, 13, 16, 19, and 22	75 days	13	1	68% vs. 8%^a^	Cancer induced in animal model
	5 mg/kg doxorubicin + 100 mg/kg cyclophosphamide single shot. 8:16 L:D	1, 4, 7, 16, 19 and 22	60 days	16	1	52% vs. 0%^a^	Cancer induced in animal model
Lévi *et al.*^53^	18 mg/kg pirarubicin i.v. single shot	3, 7, 10, 14, 19, and 23	60 days	(3, 10, 14, 23)	(19)	100% vs. 74.8%	
	25 mg/kg pirarubicin i.v. single shot	3, 7, 10, 14, 19, and 23	60 days	3, 7, 10, 14	19	89% vs. 33%^a^	
	32 mg/kg pirarubicin i.v. single shot	3, 7, 10, 14, 19, and 23	60 days	3, 10	19	∼76% vs. 0%^a^	
Mormont *et al.*^54^	25 mg/kg epirubicin i.p. single shot	2, 6, 10, 14, 18, and 22	60 days	6	18	55.7% vs. 11.5%^a^	
Peleg *et al.*^55^	15 mg/kg doxorubicin intermittent. 14:10 L:D	3, 9, 15, and 21	25 days	(9, 15)	(3)	78.0% vs. 62.5%	Significance level NR
			50 days	21	15	75.0 vs. 45.5%^a^	
Lévi *et al.*^56^	13 or 14.5 mg/kg mitoxantrone i.v. single shot	3, 7, 11, 15, 19, and 23	14 days	(3, 7, 11, 19)	(23)	100% vs. 75%	Significance level NR
	16 mg/kg mitoxantrone i.v. single shot	3, 7, 11, 15, 19, and 23	14 days	11, 15	3	100% vs. 0%^a^	
Granda *et al.*^57^	9–41.4 mg/kg doxorubicin i.v. intermittent	3, 7, 11, 15, 19, and 23	60 days	7	19	84.6% vs. 34.7^a^	Cancer induced in animal model
To *et al.*^58^	20 mg/kg doxorubicin i.p. intermittent	3, 9, 15, and 21	50 days	21	9	50.4% vs. 0%^a^	
Tabuchi *et al.*^59^	20 mg/kg doxorubicin i.p. intermittent	2, 6, 10, 14, 18, and 22	35 days	14	2	80.1% vs. 9.5%^a^	
Yang *et al.*^32^	10.2 mg/kg doxorubicin i.v. intermittent	1 and 9	60 days	(9)	(1)	100% vs. 85.4%	Significance level NR
			98 days	9	1	38.8% vs. 6.2%^a^	

The animals in all studies were exposed to a 12:12 light–dark regimen, except for two. The study by Peleg *et al.* used a 14:10 L:D regimen, and the study by Scheving *et al.* performed different experiments with two LD regimens: one with 12:12 L:D and one with 8:16 L:D. The specification includes the survival percentages in the best ZT compared to the worst ZT group.

I.p., intraperitoneal; i.v., intravenous; NR, not reported; ZT, zeitgeber time.

^a^Statistically significant difference (*P* < 0.05) either represents the statistical testing of values from best vs. worst timepoints or it represents the *P*-value in which the influence of all timepoints was assessed.

Results on body weight loss were consistent across all studies, with an optimal anthracycline administration time frame identified between ZT7 and ZT15, and the worst time frame between ZT19 and ZT3 (*Figure 2*).^32,53,55–57,60^ Comparable results were found for survival rates in the ten studies included in this analysis, yet with higher inter-study heterogeneity likely originating from different study designs (e.g. animal species, light–dark cycle, type of anthracyclines, treatment regimen, and route of administration).^32,50,51,53–59^

Importantly, only three out of the ten reported studies that included survival outcomes involved tumour-bearing rodents, allowing monitoring anticancer efficacy (*Table 4*). These studies assessed tumour shrinkage and/or survival rates in rodents with mammary adenocarcinoma or lymphatic leukaemia.^52,57,59^ In male mammary adenocarcinoma-bearing mice treated with doxorubicin at different timepoints, Granda *et al.*^57^ found that ZT7 led to the highest 60-day complete tumour inhibition rate and longest duration of tumour inhibition, corresponding with the best survival rate at this timepoint. Similarly, Halberg *et al.*^52^ reported early remission and the longest duration of remission in female rats bearing mammary adenocarcinoma when a co-regimen of doxorubicin and alkylating agent phenylalanine mustard was administered at ZT10. Additionally, Scheving *et al.*^51^ reported the highest survival rates when both doxorubicin and cyclophosphamide were jointly administered at ZT13 to leukemic mice. When changing the light–dark cycle from 12:12 to 8:16, and using female instead of male mice, the worst survival was still observed at ZT1, while the optimal timing shifted from ZT13 to ZT16.^51^

Taken together, these studies indicate that the timing of anthracycline administration relative to the light–dark cycle greatly impacts treatment efficacy and toxicity, with optimal tolerability timing between ZT7 and ZT14, and with synchronized optimal windows for treatment efficacy (*Figure 2*). However, it is important to consider that best and worst timepoints may shift according to variations in experimental design, such as type of animal model, animal sex,^51^ type of anthracycline, route of drug administration,^50^ drug dosage,^32,53,58^ concomitant drug,^55,57^ and light–dark regimen.^51,55^

### 3.2 Chronopharmacokinetics of anthracycline in rodents

To determine the optimal time for anthracycline administration, it is crucial to consider a possible diurnal variation in their pharmacokinetics, also referred to as chronopharmacokinetics. Almost all aspects of pharmacokinetics, such as drug absorption, distribution, metabolism by liver enzymes, and clearance, are circadian clock dependent.^61,62^ Doxorubicin is characterized by high plasma protein-binding and quick uptake in tissues and thus has a short half-life of the distribution phase (3–5 min). The drug is primarily metabolized in the liver by carbonyl reductase 1 and 3 (*CBR1* and *CBR3*) and aldo-keto reductase (*AKR1C*), with comparatively lower metabolism in other organs, such as the heart. These enzymes convert doxorubicin into its more cardiotoxic metabolite doxorubicinol (see Supplementary material online, *Box S1*). Interestingly, diurnal patterns in the mRNA levels of *CBR1 and CBR3* have been observed in the liver of mice,^60,63^ as well as in the human liver and heart for *CBR1*.^37^ In addition, elevated *CBR1* transcription was observed in the liver of mice treated with doxorubicin at ZT2 compared to ZT14, suggesting that this may contribute to increased production of doxorubicinol and subsequent higher cardiac toxicity.^60^

Both doxorubicin and doxorubicinol are eliminated through bile and urine, with half-lives ranging from 10 to 30 h.^64–66^ Diurnal changes in metabolism or excretion may result in variations in total drug exposure when the same doses are administered at different times. If large enough, this variation could influence toxicity and efficacy.

Two animal studies have examined anthracycline chronopharmacokinetics.^58,59^ In the study by To *et al.*,^58^ doxorubicin administration to rats at two timings with a 12-hour interval revealed a statistically significant difference in the area under the plasma time-concentration curve (AUC) of doxorubicin, with the highest AUC at ZT9 and the lowest at ZT21. Interestingly, Tabuchi *et al.*^59^ did not detect circadian variation in the AUC of doxorubicin and docetaxel in mice at time points ZT2 and ZT14. This may be explained by the chosen time points: comparing ZT2 to ZT14 may not have captured a circadian variation with its peak and trough around ZT9 and ZT21, respectively, as suggested by To *et al*.^58^ However, given the contrasting findings on cardiotoxicity and survival by To *et al.*^58^ as compared to the other reported preclinical investigations, it is possible that the 24-hour variation in pharmacokinetics found in this study does not correspond with the other animal studies.

## 4. Overview of clinical studies on chronomodulated anthracyclines

### 4.1 Cardiotoxicity

To date, nine clinical studies have been performed on the time-of-day effects of anthracycline administration in humans.^67–75^ In an RCT by Gallion *et al.*,^73^ including 342 patients with endometrial cancer, patients in the intervention arm received doxorubicin at 6 AM, and cisplatin at 6 PM, while patients in the control arm received doxorubicin at an unspecified time of day, between 9 AM and 4 PM, immediately followed by cisplatin. Interestingly, median dose of doxorubicin (246 mg/m^2^ vs. 209 mg/m^2^) was higher in the intervention arm, possibly indicating better tolerability. Despite the clinically relevant difference in anthracycline dosage, the incidence of manifest cardiotoxicity was similar in both arms. Yet, the higher dose in the chronomodulated arm may have masked the cardioprotective effect of proper drug timing. In addition, only grade 3–4 toxicities, at which cardiotoxicity has reached a symptomatic stage, were investigated. Moreover, this study design did not allow for optimal assessment of the chronomodulation effect, given the considerate amount of overlap in treatment times between the intervention and control arms. Additionally, the difference in time intervals between doxorubicin and cisplatin (11.5 h vs. 0 h) may have impacted the toxicity and efficacy outcomes.^76^ In the remaining publications studying chronomodulated anthracycline administration, cardiotoxicity was either not mentioned,^68,69,71,72^ did not occur,^58,70^ or the number of cases (range *N* = 1–3) was too limited to draw conclusions.^67,75^

### 4.2 Oncological efficacy

In most clinical studies on chronomodulated chemotherapy, oncological efficacy was unaffected by administration time (*Table 5*).^39,67,70,72,73^ So far, two RCTs have studied the effects on the efficacy of chronomodulated anthracyclines.^70,73^ In these studies, in the interventional arm, anthracyclines were administered at 6 AM and combined with cisplatin, which was administered either from 4 to 8 PM^70^ or at 6 PM.^73^ In the control arm, anthracyclines were administered at 6 PM and cisplatin from 4 to 8 AM,^70^ or at both chemotherapeutics were administered at a non-time-stipulated time point.^73^ These studies reported numerically higher response rates (73% vs. 57%)^70^ and longer progression-free survival (median, 13.0 vs. 8.0 months) in the chronomodulated arm compared to the control arm in patients with ovarian cancer.^70^ Similarly, a longer survival time (median, 13.2 vs. 11.2 months) was found in patients with endometrial cancer.^73^ While these differences in efficacy and patient survival did not reach statistical significance, both RCTs reported statistically significant reduction in haematological toxicity in the chronomodulated study arm. Similar to the previous studies, the single-arm study by Barrett *et al.*^72^ reported a favourable effect on complete response rate in patients with advanced endometrial cancer treated with doxorubicin at 6 AM and cisplatin at 6 PM. However, since a control group was lacking, results were compared to existing literature, hindering direct interpretation of the effect of the chronomodulated regimen in this patient population. Lastly, the non-randomized study by Hrushesky *et al*.^67^ did not suggest improved efficacy resulting from the chronomodulated chemotherapeutic regimen (doxorubicin: 6 AM; cisplatin: 6 PM) in a small population (*N* = 13) of bladder cancer patients.

**Table 5:** Efficacy outcome of clinical trials with chronomodulated chemotherapy including anthracyclines

First author, year	Type of cancer (stage)	Intervention group	Control group	Treatment efficacy differences
Hrushesky *et al.*^67^	Bladder cancer (III–IV)	Doxorubicin 6 AM, cisplatin 6 PM (*n* = 6)	Doxorubicin 6 PM, cisplatin 6 AM (*n* = 7)	None
Lévi *et al.*^70^	Ovarian cancer (I–IV)	Pirarubicin 6 AM, cisplatin 4–8 PM (*n* = 12)	Pirarubicin 6 PM, cisplatin 4–8 AM (*n* = 16)	Response rate (73% vs. 57%, ns); progression-free survival (median, 13.0 months vs. 8.0 months; ns)
Barret *et al.*^72^	Endometrial cancer (III–IV)	Doxorubicin 6 AM, cisplatin 6 PM (*n* = 30)	None	Higher overall response rates vs. existing literature
Gallion *et al.*^73^	Endometrial cancer (III, IV)	Doxorubicin 6 AM, cisplatin 6 PM (*n* = 170)	Doxorubicin and cisplatin non-time-stipulated (*n* = 166)	Survival time (median 13.2 months vs. 11.2 months; ns)
Kim *et al.*^75^	Diffuse large B-cell lymphoma (I–IV)	R-CHOP 8.30 AM, male (*n* = 49), female (*n* = 51)	R-CHOP 3.30 PM, male: (*n* = 74), female: (*n* = 36)	Male: No differences Female: 3-year overall survival 69.2% vs. 88.6%, due to higher haematological toxicity leading to dose reduction

Ns, non-significant; R-CHOP, rituximab, cyclophosphamide, daunorubicin, vincristine, and prednisone.

The previously mentioned studies investigated the effects of a chronomodulated regimen consisting of anthracyclines at 6 AM combined with cisplatin in the late afternoon. Although none of these studies found a statistically significant effect of the treatment schedule on efficacy measures, a trend towards an improvement in efficacy resulting from chronomodulation was observed.^70,72,73^ In contrast, the observational cohort study by Kim *et al.*^75^ found that female patients with diffuse large B-cell lymphoma experienced more haematological toxicity when receiving R-CHOP (rituximab, cyclophosphamide, daunorubicin, vincristine, and prednisone) in the morning (8:30 AM) than in the afternoon (2:30 PM). This earlier administration led to more dose reductions and, consequently, worse survival outcomes (3-year overall survival 69.2% vs. 88.6%). For males, no difference was observed regarding toxicity or survival between the morning and afternoon administration of R-CHOP. Since the RCTs by Gallion *et al.*^73^ and Lévi *et al.*,^70^ which were performed in a female population, reported lower haematological toxicity resulting from the regimen containing morning anthracyclines and afternoon cisplatin, the higher haematological toxicity in females when administering R-CHOP in the morning may be explained by the time-dependent effect of other myelosuppressive agents in the R-CHOP regimen. This stresses the need to consider the optimal administration time for each chemotherapeutic drug separately, rather than evaluating the timing of a multi-drug chemotherapeutic regimen. The finding that haematological toxicity was only timing-dependent in females may be explained by sex-specific differences in human circadian clocks.^37^ Indeed, sex-related differences in toxicity and efficacy of chronomodulated chemotherapy have previously been reported, albeit in non-anthracycline-containing regimens.^39^

Altogether, in most clinical studies investigating chronomodulated anthracycline administration, oncological efficacy was unaffected. However, due to the small number of studies and their methodological limitations, an effect on efficacy cannot be ruled out. Moreover, effects on efficacy may differ according to specific tumour characteristics. In the one study that reported lowered efficacy following R-CHOP administration in the morning, the results may have been influenced by suboptimal administration timing of individual agents and unknown confounding factors introduced by the observational study design.

### 4.3 Anthracycline chronopharmacokinetics in humans

Although no recent clinical chronopharmacokinetic studies are available, in the past, two small studies have investigated the effect of anthracycline administration time on pharmacokinetics. Canal *et al.*^71^ found that administration of doxorubicin at 9 AM resulted in a shorter half-life (mean, 12.6 h vs. 21.7 h) and a lower AUC of plasma time-concentration profiles (1742mg/h/L vs. 2503 mg/h/L) than after 9 PM administration, in a population of patients with breast cancer. The study by Eksborg *et al.*^69^ investigated the chronopharmacokinetics of epirubicin in ten patients with a gynaecological malignancy. On average, the results displayed a shorter half-life for epirubicin administered at 7 AM than at 7 PM. Even though these findings on half-life corroborate the study by Canal *et al.*,^71^ the opposite findings were reported for AUC values (higher in the morning, lower in the evening). It is unclear how this inconsistency arose since the half-life time and AUC are classically positively correlated. One explanation may lay in the small sample size, high variability, and low quality of pharmacological analysis in the study by Eksborg *et al.*^69^ Since only two administration time points were investigated in both studies, it remains unclear how anthracycline pharmacokinetics may vary over the rest of the 24 h, and an optimal time point remains to be determined. However, these clinical findings add to the evidence from animal studies, substantiating the existence of diurnal variation in anthracycline pharmacokinetics. Larger studies, comparing at least three to four dosing time points across the 24-hour span, are needed to improve our understanding of anthracycline chronopharmacokinetics.

## 5. Synchronizing circadian models for optimized anthracycline chronotherapy and cardiotoxicity mitigation

To determine the hypothesized optimal time of anthracycline administration in humans, translation of preclinical findings is needed. This translation poses a challenge, given the relative phase shift in circadian clock gene expression and differences in cardiovascular physiology between nocturnal rodents and diurnal humans. To extrapolate preclinical findings, we compared the cardiac circadian clocks of *in vitro* models and rodents to humans by aligning circadian variation of *PER2* and/or *BMAL1* mRNA expression (see Supplementary material online, *Tables S3*–5, *Figure 2*), using a novel approach based on heart-specific peripheral clock gene rhythms.

Administration of doxorubicin to cardiac cells *in vitro* demonstrated a time-dependent induction of apoptosis.^42–44^ Using the provided curves of the *BMAL1* mRNA expression per *in vitro* study, CT values were translated to clock times by matching *BMAL1* mRNA expression, measured using the Lumicycle, to human *BMAL1* expression patterns (see Supplementary material online, *Table S2*). The lowest apoptosis rates occurred at 1 PM (CT45)^42^ and 3:30 PM (CT36 and CT39),^43,44^ while the highest apoptosis rates were observed at 9:30 PM (CT18 and CT27)^42,44^ and 7 AM (CT27).^43^

Translation from rodent ZT to human clock time was performed by synchronizing peaks of *BMAL1* and *PER2* mRNA expression (see Supplementary material online, *Table S3*). *PER2* expression peaks approximately between ZT13 and ZT15 in rodent hearts,^77–80^ and 4 h after sunrise (HASR) in the human left ventricle,^81,82^ which corresponds to approximately 10 AM (based on the geographical location and season when the sun rises at 6 AM). For *BMAL1*, mRNA expression peaks between ZT23 and ZT2 in rodent hearts,^77–80,83^ and 15.4 HASR in the human left ventricle,^81,82^ at approximately 9:30 PM. Synchronizing the circadian rhythms of rodents and humans with clock time using the two distinct core clock genes (*PER2* and *BMAL1*) resulted in a 1-hour variation between the proposed clock time (see Supplementary material online, *Table S4*), which has therefore been reported, including this margin of variability. Importantly, these results were obtained from studies conducted under a 12:12 light–dark regime, the applicability of these translation strategies is limited to studies using similar light–dark cycles.

For rodents, the optimal hypothesized time frame was ZT7-14 (*Figure 2*), and translated to humans, this corresponds to 3–11 AM. We consider the echocardiographic outcomes from Yang *et al.*^32^ as particularly relevant for determining the optimal timing of anthracycline administration, as echocardiography represents the most informative method for investigating cardiotoxicity *in vivo*. They observed preserved left ventricular ejection fraction and fractional shortening at ZT9, translating to the early morning hours (5–6 AM), around which the hypothesized optimal time frame is centred. In addition, anthracycline-induced cardiac damage may be regulated through molecular mechanisms such as cellular metabolism and DNA repair, possibly involving diurnal expression of both SIRT1 and SIRT3.^23,84^ In rodents, SIRT1 and SIRT3 expression peak at ZT9 and ZT15, respectively, suggesting that the SIRT-driven alleviation of anthracycline-induced cardiotoxicity may occur primarily around 5 AM and 12 PM.^27,32,84^

**Figure 3:**
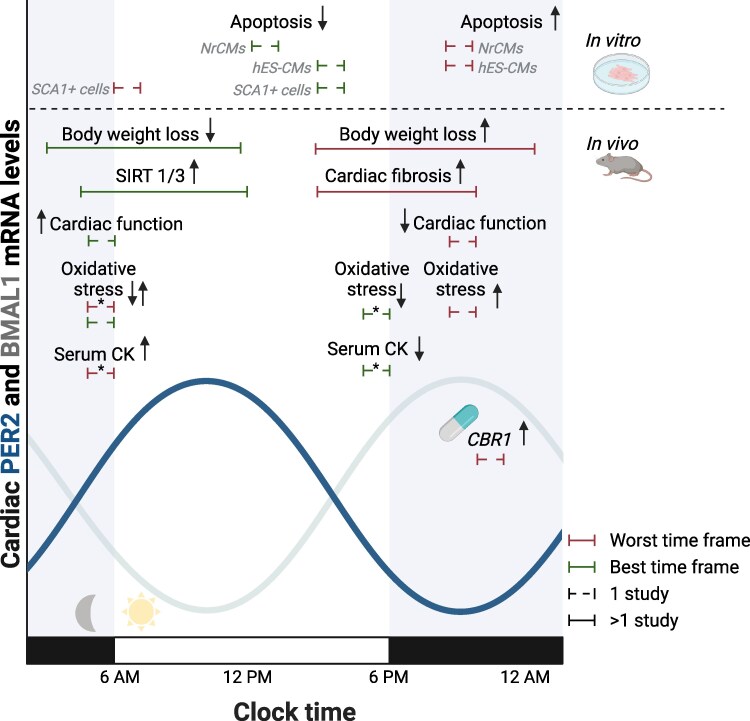
Illustration summarizing the time ranges for humans in which the heart may be least sensitive (green) and most sensitive (red) to anthracycline-induced cardiotoxicity, based on various indicators. Time ranges from *in vitro* and *in vivo* studies were translated to optimal timing in humans based on *BMAL1* and/or *PER2* expression, using data from Supplementary material online, *Tables S2*–*S4*. *Extracted from To *et al.*^58^ CBR1, Carbonyl reductase 1; CK, creatine kinase; hES-CMs, human embryonic stem cell-derived cardiomyocytes; NrCMs, neonatal rat cardiomyocytes; SCA1+ cells, stem cell antigen 1 positive cells; SIRT, sirtuins. Created in Biorender.

Taken together, we hypothesize that the optimal time of anthracycline administration lies between 3 and 11 AM (*Figure 3*). This hypothesis is based on animal studies only, since translation of *in vitro* circadian clocks to human clock time is less accurate, given the discrepancies between circadian clocks *in vitro* and *in vivo*. This is further consistent with RCTs demonstrating a trend of increased anticancer efficacy and patient survival when anthracyclines were administered at 6 AM, yet this did not reach statistical significance. More research is needed to evaluate this hypothesized optimal time frame in humans.

## 6. Discussion

Our narrative review underscores the role of circadian rhythms in anthracycline-induced cardiotoxicity and hypothesizes that the optimal administration time lies between 3 and 11 AM. Proposed mechanisms underlying this time-dependency include circadian regulation of oxidative stress response and DNA damage repair. Additionally, diurnal variations in whole-body and intracellular pharmacokinetics may play a role. Since human studies on this topic are limited, the potential advantage of chronomodulated anthracycline administration in preventing cardiotoxicity is yet to be explored.

While our hypothesized optimal time frame is based on findings from cardiotoxicity outcomes, body weight loss, and survival rate in animal studies, our proposed time frame should be interpreted with caution. Only one preclinical study has specifically evaluated cardiac function, identifying ZT9 (i.e. 5–6 AM) as the optimal administration time.^32^ In addition, heterogeneity in experimental setup across animal studies may have influenced observed optimal time points. Lastly, translation from ZT to human clock time was based on cardiac *BMAL1* and *PER2* mRNA rhythmicity. While this type of molecular alignment is an established approach in circadian biology, it has traditionally relied on central molecular clocks or systemic markers. Importantly, since rodents are nocturnal and humans are diurnal, a direct comparison—or simple inversion—of light–dark cycles across species is inappropriate. Although core clock gene phases (e.g. *BMAL1, PER2*) are conserved, their expression timing is mirrored relative to behavioural activity, meaning identical Zeitgeber times reflect opposite physiological states. By anchoring our translation on the cardiac-specific molecular rhythms, rather than behavioural phases, we aimed to provide a more biologically relevant estimate of human-equivalent treatment timing. Nevertheless, this molecular alignment does not account for interindividual variability in circadian phase or for additional physiological modulators (i.e. chronotype, exercise, diet), other than core clock gene expression. Future preclinical studies could move towards the application of diurnal models with conserved circadian clock architecture, ideally bearing tumours with defined clock phenotypes, ranging from functional to disrupted, and adopt more clinically relevant chronomodulated treatment protocols, such as prolonged low-dose intravenous anthracycline administration.

The study by To *et al.*^58^ reported discrepant findings regarding cardiotoxicity and survival compared to other studies. While it is not clear how these discrepancies arose, possible explanations may include differences in study design, such as animal model, housing and feeding conditions.

In the few animal studies that incorporated tumour-bearing animals, the best time points for efficacy improvement (ZT7–13; 3–10 AM)) aligned with the optimal time frame for cardiotoxicity, body weight loss, and survival (ZT7–14; 3–11 AM). Additionally, most clinical studies found non-significant differences resulting from timing anthracyclines in the early morning, with a trend towards improved efficacy. However, effect on efficacy may vary per malignancy type. Furthermore, clinical studies were limited and generally had a high risk of bias. Therefore, based on current evidence, it is not possible to draw definitive conclusions regarding the effects of chronomodulated anthracyclines on oncological efficacy.

Although morning administration could be beneficial in the context of anthracycline-induced cardiotoxicity, some preclinical and observational human studies have demonstrated a lower tolerance to ischaemia-induced damage during the morning.^85–87^ Whether this potential discrepancy is attributable to a difference in the cellular and molecular mediators of the damage in ischemia vs. drug-related toxicity, the chronic vs. acute response, or to differences in translatability from preclinical to clinical studies, remains to be determined in future studies.

A functional circadian clock system is imperative for the effectiveness of chronomodulated anthracycline administration in reducing cardiotoxicity. For this, interindividual differences in circadian clock function should be considered, since these may lead to variations in the optimal administration time. Patient-specific behavioural variation regarding the timing of sleep and wakefulness, exercise, and food intake may cause variations in the circadian timing system of patients.^88,89^ Also, cancer itself can induce alterations in the functional status of the circadian timing system with high interindividual variability.^90,91^ Other factors influencing circadian rhythms include sex, age, comorbidities, and various drugs.^92,93^ Notably, chronotype shifts during childhood and adolescence may modulate the effectiveness of chronotherapy. On the other hand, extensive evidence supports the existence of sex differences in the circadian timing system.^41,94^ Interestingly, sex differences in the efficacy and (non-cardiac) toxicity of non-anthracycline chemotherapeutic agents have been observed.^95,96^ Therefore, future clinical trials investigating chronomodulated anthracycline administration should assess the influence of nonmodifiable patient factors on the optimal administration time. Moreover, the effect of chronomodulated anthracycline administration may be optimized by applying clock synchronization strategies to achieve robust and aligned circadian rhythms in patients.^90,97^ These may include maintaining set times for sleeping, waking, eating, and exercising while limiting sleep-disturbing substances. In the future, chronomodulated administration of anthracyclines may be further personalized by adjusting the treatment time to each individuals’ circadian rhythms,^98–100^ as measured by molecular analysis using blood or salivary samples, or wearable technologies.^101^

Unfortunately, cancer treatment–related cardiotoxicity is not exclusively observed in anthracycline treatment. Other systemic anticancer treatments (e.g. fluoropyrimidines, trastuzumab, osimertinib) and radiotherapy have proven cardiotoxic side effects, although on a smaller scale than anthracyclines and via other mechanisms.^102^ Additionally, indications for immunotherapy are rapidly increasing, and subsequent life-threatening immune-related cardiac toxicities, such as immune checkpoint inhibitor myocarditis, are frequently observed.^103,104^ Several studies suggest that treatment timing may influence efficacy and toxicity. For example, chronomodulated administration of immune checkpoint inhibitors has been associated with improved survival,^105^ and chronoradiation has shown benefits in head and neck cancer.^106^ However, cardiac outcomes have not been assessed in these studies. Moreover, most chronotherapy research has focused on monotherapies, whereas standard cancer regimens often comprise multiple agents with potentially conflicting optimal timing windows. Future studies should therefore explore circadian-informed strategies in combination therapies, ideally supported by computational models such as physiologically based pharmacokinetic simulations^107^ or machine-learning-based models to simulate timing-dependent interactions and guide personalized treatment scheduling.

Although the preclinical studies have shown promising evidence supporting the potential application of chronomodulated anthracycline administration for mitigating cardiotoxicity, a significant translational gap remains, as much of the foundational evidence originates from studies conducted decades ago. The integration of chronomodulated chemotherapy into standard clinical practice is impeded by several significant challenges, some of which could be addressed through further preclinical investigation. Critical knowledge gaps persist in defining the optimal timing and dosage of various chemotherapy agents and treatment regimens, identifying patient subgroups (e.g. based on chronotype, sex, cancer subtype and baseline cardiovascular risk), determining which cardiotoxicity phenotypes (i.e. acute vs. late-onset heart failure, pericardial disease, and arrhythmias) are most likely to benefit from chronotherapy, and elucidating the molecular mechanisms underlying its effects. Modern preclinical models, including in silico systems, patient-specific induced pluripotent stem cell-derived cardiomyocytes, and organ-on-a-chip platforms, present valuable tools for investigating these questions. Addressing these gaps would facilitate the refinement of chronotherapy, enabling its more precise and effective application in clinical settings.

In addition to these scientific barriers, substantial logistical and financial obstacles hinder the clinical translation of chronomodulated chemotherapy. Unlike novel pharmacological agents, chronotherapy does not require the development of new drugs, thereby reducing its appeal to pharmaceutical companies as a potential investment. As a result, securing alternative sources of trial funding from governmental bodies or healthcare insurers is imperative. Moreover, the adoption of (personalized) time-dependent chemotherapy scheduling would necessitate extensive institutional restructuring, likely encountering resistance due to the required modifications to hospital workflows. Raising awareness of the potential benefits of chronomodulation is therefore essential, though it remains a significant challenge, particularly within an emerging field such as cardio-oncology. Overcoming these barriers through increased research investment, interdisciplinary collaboration, and broader clinical engagement is crucial to realizing the full potential of chronomodulated cardiotoxic anticancer therapies.

In conclusion, this review provides a summary of the available evidence supporting the application of chronomodulated anthracycline administration to reduce cardiotoxicity. Translation of these findings to human clock time reveals a hypothetical optimal administration time between 3 and 11 AM, which initiates the process of bridging the translational gap. Given the limitations discussed, we consider it premature to prescribe an exact timing for treatment regimens involving anthracycline at this stage. Nevertheless, in patients at high risk of cardiotoxicity, particularly those receiving cumulative anthracycline doses, circadian-timed drug administration remains a biologically plausible and promising strategy that warrants prospective clinical investigation. Dedicated clinical trials are needed to validate the proposed timing, evaluate the application of chronomodulated anthracycline administration, assessing the effect on cardiotoxicity while ensuring that oncological efficacy is not compromised. To further investigate this intervention, a study population consisting of cancer patients receiving treatment in an adjuvant setting, with a high risk of developing cardiotoxicity, would be preferred. An RCT with an adequate sample size, comparing at least three treatment time points, would be most suitable for identifying the optimal administration time for anthracyclines, which could lead to a substantial benefit for the patients.

## Supplementary Material

cvaf179_Supplementary_Data

## Data Availability

The data underlying this article are available in the article and in its online supplementary material.
