# Supplementary material for: Chronotherapy as a novel strategy to limit anthracycline-induced cardiotoxicity
Source: Cardiovasc Res. 2025 Oct 6;121(14):2144–56. doi: 10.1093/cvr/cvaf179 (PMC12638736; doi:10.1093/cvr/cvaf179)
Supplement: cvaf179_Supplementary_Data [file cvaf179_supplementary_data.docx]

**Supplemental material**

**Chronotherapy as a novel strategy to limit anthracycline-induced cardiotoxicity**

**Content**

**Supplemental results**

### BOX 1: Doxorubicinol: a metabolite mediating myocardial damage – page 2-3

**Supplemental tables**

Supplemental Table 1: Search query – page 4

Supplemental Table 2: *In vitro* studies assessing the apoptosis rates aligned with *BMAL1* mRNA expression– page 5

Supplemental Table 3: Timing of peak mRNA expression of *PER2* and *BMAL1* for synchronizing circadian models – page 6

Supplemental Table 4: Synchronizing the circadian rhythms of rodents and humans to clock time – page 7

Supplemental Table 5: Data extraction from Figure 1 - page 8-9

**Supplemental figures**

Supplemental Figure 1 - Flow diagram of the study selection – page 10

**Supplemental methods**

Search strategy, selection criteria, data extraction, studies excluded based on full text – page 11-13

**Supplemental references -** page 14-16

**Supplemental results**

### **BOX 1**

| **Doxorubicinol: a metabolite mediating myocardial damage**Next to pharmacokinetics in general, drug metabolism may be involved in cardiotoxicity. In doxorubicin induced cardiotoxicity, the doxorubicin metabolite doxorubicinol is hypothesized to cause higher cardiotoxicity than doxorubicin.^1,2^ In the heart, the most abundant metabolizing enzyme is carbonyl reductase 1 (CBR1).^3^ The half-life of CBR1 is relatively short (9.4 hours),^4^ which may lead to significant fluctuations in its levels and, consequently, its ability to metabolize doxorubicin. Human CBR1 and CBR3 knockout induced pluripotent stem cell-derived cardiomyocytes, showed reduced production of reactive oxygen species following doxorubicin exposure *in vitro*.^5^ Moreover, pharmacological inhibition of CBR1 has been shown to synergize with doxorubicin in inducing tumor cell death, while concurrently mitigating cardiotoxicity, as demonstrated by enhanced echocardiographic parameters and reduced cardiac damage.^6^ Conversely, the transgenic expression of human CBRs in mice led to increased formation of doxorubicinol, exacerbating cardiotoxicity and hastening cardiac death.^7^Additionally, the relative concentration of doxorubicinol to doxorubicin is higher in cardiac tissue and accumulates over time.^8,9^ Importantly, doxorubicinol is more hydrophilic compared to doxorubicin, which reduced its permeability through cell membranes and extend displays reduced permeability to doxorubicinol, resulting in an extended residency period within cardiomyocytes.^10,11^ These findings suggest that doxorubicin-induced cardiotoxicity is partially attributable to its metabolite, doxorubicinol. Estimating the respective contributions of doxorubicin and doxorubicinol to this cardiotoxicity is challenging since doxorubicin’s concentration is higher in cardiac tissue whereas doxorubicinol is more cardiotoxic. Understanding the temporal dynamics of doxorubicin conversion to doxorubicinol is crucial for reducing cardiotoxicity through chronotherapy. |
| --- |

**Supplemental tables**

**Supplemental Table 1. Search query**

("Circadian"[Title/Abstract] OR "clock*"[Title/Abstract] OR "biological rhythm"[Title/Abstract] OR "biological rhythms"[Title/Abstract] OR "chronomodulat*"[Title/Abstract] OR "melatonin"[Title/Abstract] OR "chrono modulated"[Title/Abstract] OR (("chrono"[All Fields] OR "chronos"[All Fields]) AND "modulate"[Title/Abstract]) OR "chrono modulation"[Title/Abstract] OR "chronotherap*"[Title/Abstract] OR "chrono therapy"[Title/Abstract] OR "chrono therapies"[Title/Abstract] OR "chrono therapeutic"[Title/Abstract] OR "chronobiolog*"[Title/Abstract] OR "chrono biology"[Title/Abstract] OR "chrono biological"[Title/Abstract] OR "chronopharmacologic*"[Title/Abstract] OR "twenty four hour"[Title/Abstract] OR "Nycthemeral"[Title/Abstract] OR "Nyctohemeral"[Title/Abstract] OR "Diurnal"[Title/Abstract] OR "chronochemotherap*"[Title/Abstract] OR "periodicit*"[Title/Abstract] OR ("Chronotherapy"[MeSH Terms] OR "Chronobiology Phenomena"[MeSH Terms])) AND ("anthracyclin*"[Title/Abstract] OR "doxorubicin"[Title/Abstract] OR "adriamycin"[Title/Abstract] OR "pirarubicin"[Title/Abstract] OR "daunorubicin"[Title/Abstract] OR "epirubicin"[Title/Abstract] OR "idarubicin"[Title/Abstract] OR "aclarubicin"[Title/Abstract] OR "carubicin"[Title/Abstract] OR "nogalamycin"[Title/Abstract] OR "plicamycin"[Title/Abstract] OR "anthracyclines"[MeSH Terms])

**Supplemental Table 2**. *In vitro* studies assessing the apoptosis rates aligned with *BMAL1* mRNA expression measured using Lumicycle.

| **Apoptosis rate** | | **NrCMs**^12^ | **hES-CMs**^13^ | **SCA1+ cells**^14^ |
| --- | --- | --- | --- | --- |
| **High** | Circadian time  *BMAL1* expression | 27  *Peak* | 18  *Peak* | 27  *Intersect - Trough* |
| **Low** | Circadian time  *BMAL1* expression | 45  *Trough - intersect* | 36  *Intersect (acrophase)* | 39  *Intersect (acrophase)* |
| *BMAL1*, basic helix-loop-helix ARNT like 1; hES-CMs, Human embryonic Stem cell-derived cardiomyocytes; NrCMs, Neonatal rat cardiomyocytes; SCA1+ cells, Stem cell antigen 1 positive cells. | | | | |

**Supplemental Table 3.** Timing of peak mRNA expression of *PER2* and *BMAL1* for synchronizing circadian models.

|  | **Peak *PER2*** | **Peak *BMAL1*** | **Reference** |
| --- | --- | --- | --- |
| **Humans** |  |  |  |
| *HASR* | 3.63 - 4.51 | 14.47 - 16.25 | ^15^ |
| *Clock time** | 9:40 AM - 10:30 AM | 8:28 PM - 10:15 PM | ^15,16^ |
| **Rodents** |  |  |  |
| *ZT* | ZT13-15 | ZT23-2 | ^17–20^ |
| *Clock time** | 7:00 PM – 9:00 PM | 3:00 AM – 8:00 AM |  |
| *****based on the geographical location and season when the sun rises at 6 AM. *BMAL1*, basic helix-loop-helix ARNT like 1; HASR: hours after sun rise; PER2, period circadian regulator 2; ZT: zeitgeber time. | | | |

| **Clock time** | 12 AM | 1 AM | 2 AM | 3 AM | 4 AM | 5 AM | 6 AM | 7 AM | 8 AM | 9 AM | 10 AM | 11 AM | 12 PM | 1 PM | 2 PM | 3 PM | 4 PM | 5 PM | 6 PM | 7 PM | 8 PM | 9 PM | 10 PM | 11 PM |  |
| --- | --- | --- | --- | --- | --- | --- | --- | --- | --- | --- | --- | --- | --- | --- | --- | --- | --- | --- | --- | --- | --- | --- | --- | --- | --- |
| **Human (HASR)** |  |  |  |  |  |  |  |  |  |  |  |  |  |  |  |  |  |  |  |  |  |  |  |  |  |
| *PER2* | 18 | 19 | 20 | 21 | 22 | 23 | 0 | 1 | 2 | 3 | **4*** | 5 | 6 | 7 | 8 | 9 | 10 | 11 | 12 | 13 | 14 | 15 | 16 | 17 |  |
| *BMAL1* | 18 | 19 | 20 | 21 | 22 | 23 | 0 | 1 | 2 | 3 | 4 | 5 | 6 | 7 | 8 | 9 | 10 | 11 | 12 | 13 | 14 | **15*** | 16 | 17 |  |
| **Rodent (ZT)** |  |  |  |  |  |  |  |  |  |  |  |  |  |  |  |  |  |  |  |  |  |  |  |  |  |
| *PER2* | 4 | 5 | 6 | 7 | 8 | 9 | 10 | 11 | 12 | 13 | **14*** | 15 | 16 | 17 | 18 | 19 | 20 | 21 | 22 | 23 | 0 | 1 | 2 | 3 |  |
| *BMAL1* | 3 | 4 | 5 | 6 | 7 | 8 | 9 | 10 | 11 | 12 | 13 | 14 | 15 | 16 | 17 | 18 | 19 | 20 | 21 | 22 | 23 | **0*** | **1*** | 2 |  |
|  |  |  |  |  |  |  |  |  |  |  |  |  |  |  |  |  |  |  |  |  |  |  |  |  |  |
| *****Time of peak expression. *BMAL1*, basic helix-loop-helix ARNT like 1; HASR: hours after sun rise; PER2, period circadian regulator 2; ZT: zeitgeber time. | | | | | | | | | | | | | | | | | | | | | | | | | |

**Supplemental Table 4. Synchronizing the circadian rhythms of rodents and humans to clock time.** Human and rodent *PER2* and *BMAL1* mRNA expression peaks were synchronized. Clock time was calculated based on the assumption that 0 hours after sunrise corresponds to 6

**Supplemental Table 5.** Data extraction from figure 1. Outcomes from *in vitro* studies using Circadian time (CT) were aligned with human clock times using *BMAL1* mRNA expression patterns. Translation from *in vitro* CT to clock times are indicative, as *in vitro* clocks are different than clocks in whole-body organisms, hindering precise translation. Outcomes from rodent studies were translated to clock times using *BMAL1* and *PER2* mRNA expression peaks, in accordance with Supplementary Table 4.

|  |  | **CT** | **Clock Time** | **Ref** |
| --- | --- | --- | --- | --- |
| ***In vitro*** | |  |  |  |
|  | Highest apoptosis NrCMs | 27 | 9:30 PM | ^12^ |
|  | Highest apoptosis hES-CMs | 18 | 9:30 PM | ^13^ |
|  | Highest apoptosis SCA1+ cells | 27 | 7 AM | ^14^ |
|  | Lowest apoptosis NrCMs | 45 | 1 PM | ^12^ |
|  | Lowest apoptosis hES-CM | 36 | 3:30 PM | ^13^ |
|  | Lowest apoptosis SCA1+ cells | 39 | 3:30 PM | ^14^ |
| **Rodent studies** | |  |  |  |
| *Cardiotoxicity* | |  |  |  |
|  | Preserved cardiac function [LVEF, FS, LVW/TL] | 9 | 5 – 6 AM | ^21^ |
|  | Impaired cardiac function | 1 | 9 – 10 PM | ^21^ |
|  | Cardiac fibrosis | 19 – 1 | 3 – 10 PM | ^21,22^ |
|  | Oxidative stress high [H_2_O_2_ levels, MnSOD activity] | 1 | 9 – 10 PM | ^21^ |
|  | Oxidative stress low [H_2_O_2_ levels, MnSOD activity] | 9 | 5 – 6 AM | ^21^ |
|  | Oxidative stress high [MDA levels] | 9 | 5 – 6 AM | ^23^ |
|  | Oxidative stress low [MDA levels] | 21 | 5 – 6 PM | ^23^ |
|  | Serum CK high | 9 | 5 – 6 AM | ^23^ |
|  | Serum CK low | 21 | 5 – 6 PM | ^23^ |
|  | SIRT1 peak | 15 | 11 AM – 12 PM | ^24^ |
|  | SIRT3 peak | 9 | 5 – 6 AM | ^21,25^ |
| *Other* | |  |  |  |
|  | Highest toxicity [body weight loss] | 19 – 3 | 3 PM – 12 AM | ^21,22,26–29^ |
|  | Lowest toxicity [body weight loss] | 7 – 15 | 3 AM – 12 PM | ^21,22,26–29^ |
|  | Highest induction of *CBR1* expression in the liver | 2 | 10 – 11 PM | ^26^ |
| CBR1, Carbonyl reductase 1; CK, Creatine kinase; FS, Fractional shortening; hES-CMs, Human embryonic Stem cell-derived cardiomyocytes; LVEF, Left ventricular ejection fraction; LVW/TL, Left ventricular weight/tibial length; MDA, Malondialdehyde; MnSOD, Manganese superoxide dismutase; NrCMs, Neonatal rat cardiomyocytes; ref, Reference; SCA1+ cells, Stem cell antigen 1 positive cells; SIRT, Sirtuin; ZT, Zeitgeber Time. | | | | |

**Supplemental figures**

**Supplemental Figure 1.** Flow diagram of the study selection.


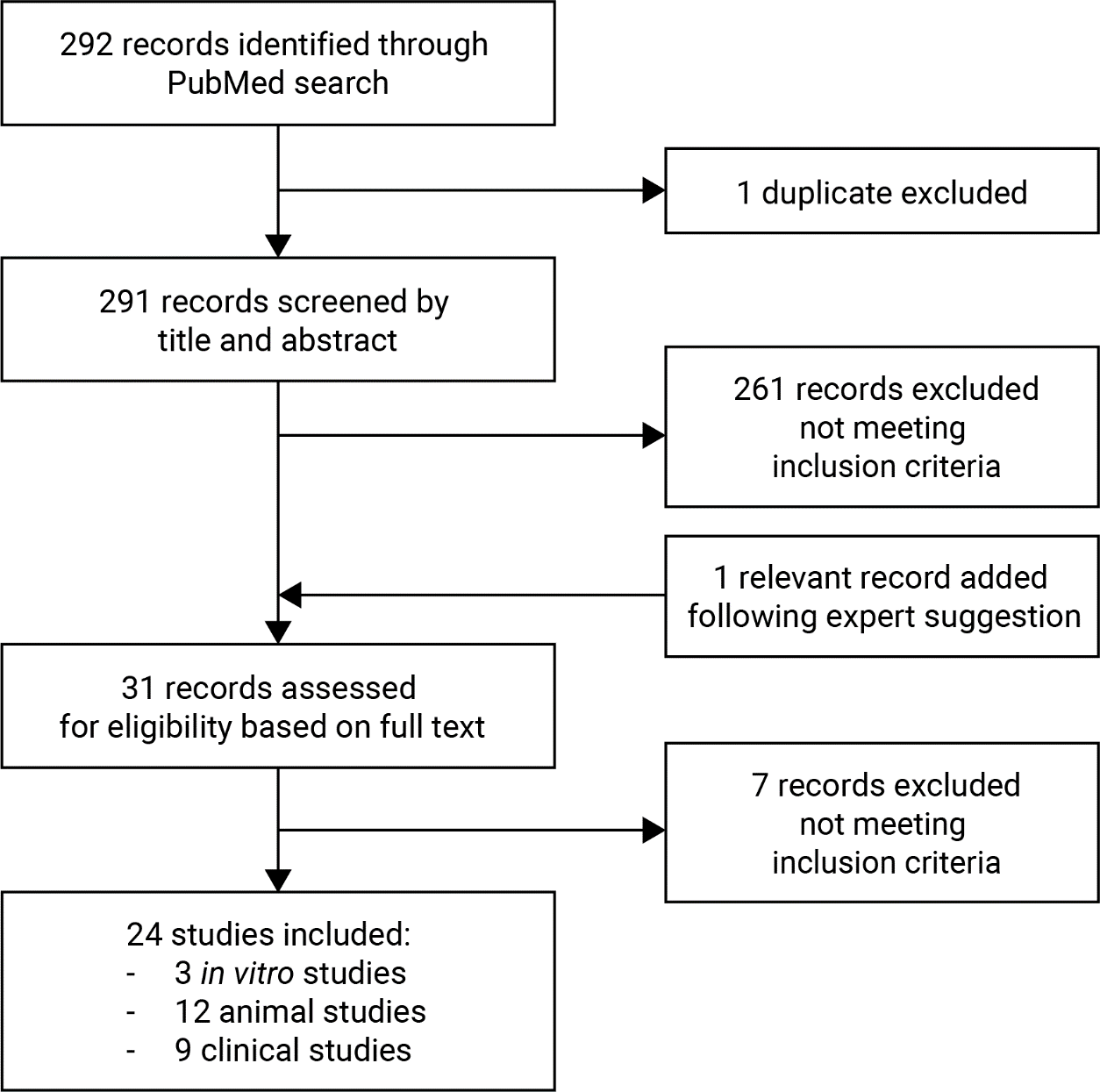


**Supplemental methods**

Search strategy

In this narrative review, we included pre-clinical and clinical studies that evaluated the toxicity and efficacy of chronomodulated anthracycline-based cancer therapies, limited to publications in English, German, or Dutch. A comprehensive PubMed search was conducted on May 4, 2023, and updated on October 22, 2024, without a restriction on publication date. The search strategy employed synonyms and related terms for ’Circadian rhythms’ and ‘Anthracyclines’ and included the MeSH terms for ‘Chronotherapy’, ‘Chronobiology phenomena’, and ‘Anthracyclines’ (**Supplemental Table 1**). Duplicate records were removed, and titles, abstracts, and full texts were independently screened using the selection criteria by MIP and IRK with Rayyan, a web-based tool for reviews (Rayyan Systems Inc., Cambridge, USA).

Selection criteria

Preclinical studies were included given that they compared the effects of administering anthracyclines at varying time points across the 24-hour cycle. At least one of the following outcome measures had to be reported: cardiotoxicity indicators, body weight, survival rate, and tumor regression.

Clinical studies investigating chronomodulated anthracycline regimes in cancer patients were included (**Supplemental Figure 1**).

Data extraction

Data from the included preclinical studies were extracted by IRK, while MIP extracted the results from the clinical studies. The following parameters were collected:

- Publication information: author, year, title, journal.
- Methodology and results
  - *In vitro* studies:

General characteristics: cell type, species;

Methods: synchronization method, method used for assessing circadian gene expression;

Treatment: drug type, dose, exposure time;

Outcome: highest/lowest apoptosis rate.

- - Animal studies:

Animal characteristics: species, sex, age, and weight of the animals, number of animals used, type of (introduced) disease and its induction method, follow-up time;

Treatment characteristics: light-dark regimen, timings of administration, type and dose of chemotherapeutic drugs, route of administration;

Outcomes: cardiotoxicity indicators, body weight, survival, tumor regression and associated best time point(s) and worst time point(s).

- - Clinical studies:

Study population: sex, age, malignancy type and stage;

Treatment: chemotherapeutic regimen, timing of administration;

Outcomes: overall survival, progression free survival, response rate, or any other efficacy indicator, left ventricular ejection fraction, heart failure, or any other cardiotoxicity indicator.

Studies excluded based on full text assessment

1. Boor PJ. Cardiac glutathione: diurnal rhythm and variation in drug-induced cardiomyopathy. *Res Commun Chem Pathol Pharmacol*. 1979;24(1):27-36.
2. Burns ER. Circadian biological time influences the effect adriamycin has on DNA synthesis in mouse bone marrow, ileum and tongue but not Ehrlich ascites carcinoma. *Oncology*. 1985;42(6):384-387.
3. Sothern RB, Lévi F, Haus E, Halberg F, Hrushesky WJ. Control of a murine plasmacytoma with doxorubicin-cisplatin: dependence on circadian stage of treatment. *J Natl Cancer Inst*. 1989;81(2):135-145.
4. Oustrin J, Sqalli A. Chronopharmacocinétique de la doxorubicine (adriamycine) [Chronopharmacokinetics of doxorubicin (adriamycin)]. *Ann Pharm Fr*. 1988;46(3):163-170.
5. Alagol H, Dinc S, Basgut B, Abacioglu N. Temporal variation in the recovery from impairment in adriamycin-induced wound healing in rats. *J Circadian Rhythms*. 2007;5:6.
6. Arif IS, Hooper CL, Greco F, Williams AC, Boateng SY. Increasing doxorubicin activity against breast cancer cells using PPARγ-ligands and by exploiting circadian rhythms. *Br J Pharmacol*. 2013;169(5):1178-1188.
7. Mitchell MI, Engelbrecht AM. Circadian Rhythms and Breast Cancer: The Role of Per2 in Doxorubicin-Induced Cell Death. *J Toxicol*. 2015:392360.

**Supplemental References**

1. Boucek RJ, Olson RD, Brenner DE, Ogunbunmi EM, Inui M, Fleischer S. The major metabolite of doxorubicin is a potent inhibitor of membrane-associated ion pumps. A correlative study of cardiac muscle with isolated membrane fractions. *J Biol Chem* 1987 **262**(33) :15851–6.

2. Mushlin PS, Cusack BJ, Boucek RJ, Andrejuk T, Li X, Olson RD. Time-related increases in cardiac concentrations of doxorubicinol could interact with doxorubicin to depress myocardial contractile function. *Br J Pharmacol* 1993 **110**(3) :975–82.

3. Bains OS, Szeitz A, Lubieniecka JM, Cragg GE, Grigliatti TA, Riggs KW, Reid RE. A Correlation between Cytotoxicity and Reductase-Mediated Metabolism in Cell Lines Treated with Doxorubicin and Daunorubicin. *J Pharmacol Exp Ther* 2013 **347**(2) :375–87.

4. Christiano R, Nagaraj N, Fröhlich F, Walther TC. Global proteome turnover analyses of the Yeasts S. cerevisiae and S. pombe. *Cell Rep* 2014 **9**(5) :1959–65.

5. Fonoudi H, Jouni M, Cejas RB, Magdy T, Blancard M, Ge N, Shah DA, Lyra-Leite DM, Neupane A, Gharib M, Jiang Z, Sapkota Y, Burridge PW. Functional Validation of Doxorubicin-Induced Cardiotoxicity-Related Genes. *JACC CardioOncol* 2024 **6**(1) :38–50.

6. Jo A, Choi TG, Jo YH, Jyothi KR, Nguyen MN, Kim JH, Lim S, Shahid M, Akter S, Lee S, Lee KH, Kim W, Cho H, Lee J, Shokat KM, Yoon KS, Kang I, Ha J, Kim SS. Inhibition of Carbonyl Reductase 1 Safely Improves the Efficacy of Doxorubicin in Breast Cancer Treatment. *Antioxid Redox Signal* 2017 **26**(2) :70–83.

7. Forrest KML, Al-Sarraj S, Sewry C, Buk S, Tan SV, Pitt M, Durward A, McDougall M, Irving M, Hanna MG, Matthews E, Sarkozy A, Hudson J, Barresi R, Bushby K, Jungbluth H, Wraige E. Infantile onset myofibrillar myopathy due to recessive CRYAB mutations. *Neuromuscular Disorders* 2011 **21**(1) :37–40.

8. Peters JH, Gordon GR, Kashiwase D, Acton EM. Tissue distribution of doxorubicin and doxorubicinol in rats receiving multiple doses of doxorubicin. *Cancer Chemother Pharmacol* 1981 **7**(1) :65–9.

9. Cusack BJ, Young SP, Driskell J, Olson RD. Doxorubicin and doxorubicinol pharmacokinetics and tissue concentrations following bolus injection and continuous infusion of doxorubicin in the rabbit. *Cancer Chemother Pharmacol* 1993 **32**(1) :53–8.

10. Zeng X, Cai H, Yang J, Qiu H, Cheng Y, Liu M. Pharmacokinetics and cardiotoxicity of doxorubicin and its secondary alcohol metabolite in rats. *Biomed Pharmacother* 2019 **116** :108964.

11. Minsley GE, Warren DW, Hairfield WM. The effect of cleft palate speech aid prostheses on the nasopharyngeal airway and breathing. *J Prosthet Dent* 1991 **65**(1) :122–6.

12. du Pré BC, Dierickx P, Crnko S, Doevendans PA, Vos MA, Geijsen N, Neutel D, van Veen TAB, van Laake LW. Neonatal rat cardiomyocytes as an in vitro model for circadian rhythms in the heart. *J Mol Cell Cardiol* 2017 **112** :58–63.

13. Dierickx P, Vermunt MW, Muraro MJ, Creyghton MP, Doevendans PA, van Oudenaarden A, Geijsen N, Van Laake LW. Circadian networks in human embryonic stem cell-derived cardiomyocytes. *EMBO Rep* 2017 **18**(7) :1199–212.

14. Du Pré BC, Demkes EJ, Feyen DAM, Dierickx P, Crnko S, Kok BJM, Sluijter JPG, Doevendans PA, Vos MA, Van Veen TAB, Van Laake LW. SCA1+ Cells from the Heart Possess a Molecular Circadian Clock and Display Circadian Oscillations in Cellular Functions. *Stem Cell Reports* 2017 **9**(3) :762–9.

15. McTiernan CF, Lemster BH, Bedi KC, Margulies KB, Moravec CS, Hsieh PN, Shusterman V, Saba S. Circadian Pattern of Ion Channel Gene Expression in Failing Human Hearts. *Circ Arrhythm Electrophysiol* 2021 **14**(1) :e009254.

16. Leibetseder V, Humpeler S, Svoboda M, Schmid D, Thalhammer T, Zuckermann A, Marktl W, Ekmekcioglu C. Clock genes display rhythmic expression in human hearts. *Chronobiol Int* 2009 **26**(4) :621–36.

17. Szántóová K, Zeman M, Veselá A, Herichová I. Effect of phase delay lighting rotation schedule on daily expression of per2, bmal1, rev-erbα, pparα, and pdk4 genes in the heart and liver of Wistar rats. *Mol Cell Biochem* 2011 **348**(1–2) :53–60.

18. Herichová I, Šoltésová D, Szántóová K, Mravec B, Neupauerová D, Veselá A, Zeman M. Effect of angiotensin II on rhythmic per2 expression in the suprachiasmatic nucleus and heart and daily rhythm of activity in Wistar rats. *Regul Pept* 2013 **186** :49–56.

19. Young ME, Razeghi P, Taegtmeyer H. Clock genes in the heart: characterization and attenuation with hypertrophy. *Circ Res* 2001 **88**(11) :1142–50.

20. Durgan DJ, Hotze MA, Tomlin TM, Egbejimi O, Graveleau C, Abel ED, Shaw CA, Bray MS, Hardin PE, Young ME. The intrinsic circadian clock within the cardiomyocyte. *Am J Physiol Heart Circ Physiol* 2005 **289**(4) :H1530-1541.

21. Yang N, Ma H, Jiang Z, Niu L, Zhang X, Liu Y, Wang Y, Cheng S, Deng Y, Qi H, Wang Z. Dosing depending on SIRT3 activity attenuates doxorubicin-induced cardiotoxicity via elevated tolerance against mitochondrial dysfunction and oxidative stress. *Biochem Biophys Res Commun* 2019 **517**(1) :111–7.

22. Levi F, Mechkouri M, Roulon A, Bailleul F, Lemaigre G, Reinberg A, Mathe G. Circadian rhythm in tolerance of mice for the new anthracycline analog 4’-O-tetrahydropyranyl-adriamycin (THP). *Eur J Cancer Clin Oncol* 1985 **21**(10) :1245–51.

23. To H, Ohdo S, Shin M, Uchimaru H, Yukawa E, Higuchi S, Fujimura A, Kobayashi E. Dosing time dependency of doxorubicin-induced cardiotoxicity and bone marrow toxicity in rats. *J Pharm Pharmacol* 2003 **55**(6) :803–10.

24. Pizarro A, Hayer K, Lahens NF, Hogenesch JB. CircaDB: a database of mammalian circadian gene expression profiles. *Nucleic Acids Res* 2013 **41**(Database issue) :D1009-1013.

25. Peek CB, Affinati AH, Ramsey KM, Kuo HY, Yu W, Sena LA, Ilkayeva O, Marcheva B, Kobayashi Y, Omura C, Levine DC, Bacsik DJ, Gius D, Newgard CB, Goetzman E, Chandel NS, Denu JM, Mrksich M, Bass J. Circadian clock NAD+ cycle drives mitochondrial oxidative metabolism in mice. *Science* 2013 **342**(6158) :1243417.

26. Borniger JC, Walker Ii WH, Gaudier-Diaz MM, Stegman CJ, Zhang N, Hollyfield JL, Nelson RJ, DeVries AC. Time-of-Day Dictates Transcriptional Inflammatory Responses to Cytotoxic Chemotherapy. *Sci Rep* 2017 **7** :41220.

27. Peleg L, Ashkenazi IE, Carlebach R, Chaitchik S. Time-dependent toxicity of drugs used in cancer chemotherapy: separate and combined administration. *Int J Cancer* 1989 **44**(2) :273–5.

28. Lévi F, Tampellini M, Metzger G, Bizi E, Lemaigre G, Hallek M. Circadian changes in mitoxantrone toxicity in mice: relationship with plasma pharmacokinetics. *Int J Cancer* 1994 **59**(4) :543–7.

29. Granda TG, Filipski E, D’Attino RM, Vrignaud P, Anjo A, Bissery MC, Lévi F. Experimental chronotherapy of mouse mammary adenocarcinoma MA13/C with docetaxel and doxorubicin as single agents and in combination. *Cancer Res* 2001 **61**(5) :1996–2001.
